# Traditional Food Items in Ogimi, Okinawa: l-Serine Content and the Potential for Neuroprotection

**DOI:** 10.1007/s13668-017-0191-0

**Published:** 2017-02-07

**Authors:** Paul Alan Cox, James S. Metcalf

**Affiliations:** Brain Chemistry Labs, Institute for Ethnomedicine, Box 3464, Jackson, WY 83001 USA

**Keywords:** l-Serine, Dietary amino acids, Tangle diseases, Alzheimer’s disease, ALS, Neuroprotection

## Abstract

**Purpose of Review:**

Ogimi village is renowned for its aging population. We sought to determine if the l-serine content of their diet could account for their neurological health.

**Recent Findings:**

The most frequently consumed food items, including tofu and seaweeds, are rich in the dietary amino acid l-serine. l-serine content of the Ogimi diet >8 grams/day for Ogimi women significantly exceeds the average American dietary intake of 2.5 grams/day for women >70 years old.

**Summary:**

Our hypothesis that the high l-serine content of the Ogimi diet is related to the paucity of tangle diseases among villagers is buttressed by *in vivo* results with non-human primates where dietary l-serine slowed development of neurofibrillary tangles and β-amyloid plaques by up to 85% and a human clinical trial finding that l-serine at 15 grams/day twice daily slows functional decline in ALS patients. Analysis of the Ogimi diet suggests that l-serine should be evaluated for therapeutic potential as a neuroprotective agent.

## Introduction

The population of Okinawa has achieved considerable fame for the overall longevity of its population, with Okinawan women having an average life expectancy of 85.08 years, exceeding the average Japanese women’s life expectancy of 83.99 years [1, 2]. Within Okinawa, the remote Ogimi village at the extreme north of the island has achieved the title of “longevity village” for its high number of centenarians. Despite considerable attention having been given to the Okinawan diet [3], there has not been an ethnobotanical survey performed for Ogimi village regarding dietary preferences.

Within the Okinawan political context, Ogimi village is a clear outlier due to its remoteness and its cultural isolation. Remarkably, the indigenous people in Ogimi have maintained their own language which is not understood by other Okinawans. Ogimi villagers learn Japanese in school, but maintain a distinct culture and religion.

The Ogimi diet has unique elements based on marine algae. Ogimi villagers who move to the Okinawan capital of Naha ask family members to supply them with those seaweeds. To explore a possible neuroprotective function for l-serine, we surveyed the Ogimi diet and analyzed the most frequently consumed items for l-serine content.

## l-Serine as a Potential Neuroprotective Agent

l-Serine is abundant in human diets. The mean concentration in blood plasma is 0.12 mM and is not reduced in elderly Okinawans [4], while in intracellular tissue the mean is 0.98 mM [5]. l-Serine is produced within the central nervous system [6] and is essential for cell cultures and cell proliferation [7]. l-Serine, a precursor for l-glycine, is produced from 3-phosphoglycerate. Transport of l-serine across the blood-brain barrier is via the sodium-dependent system A and the sodium-independent alanine, serine, and cysteine preferring system (ASC) [8–10]. Made by astrocytes and neurons [11, 12], l-serine is a precursor to phosphoglycerols, sphingolipids, sulfur amino acids, and d-serine which serves as a cofactor at NMDA receptors. It is enzymatically degraded by serine dehydratase, with NH_3_ and pyruvate as the end nitrogen and carbon metabolites, respectively [13].

A deficiency in l-serine can trigger the genetic disease 3-phosphoglycerate dehydrogenase deficiency and 3-phosphoserine phosphatase deficiency [14]. l-Serine is currently in a phase I clinical trial as a treatment for hereditary sensory autonomic neuropathy type 1 [15].

Indications that l-serine may be neuroprotective came from studies of human neuronal cell culture in which protein aggregation and cell apoptosis was triggered by the cyanobacterial toxin β-*N*-methylamino-l-alanine (BMAA) [16]. BMAA contaminated the diet of the Chamorro people of Guam [17]. Increasing l-serine in neuronal cell culture prevents BMAA misincorporation [18•].

Vervet monkeys with chronic dietary exposure to BMAA developed neurofibrillary tangles and plaques immunopositive for phosphotau and β-amyloid deposits consistent with Guam ALS/PDC [19••, 20]. Based on both *in vitro* and *in vivo* indications of a neuroprotective function for l-serine, an FDA-approved phase I clinical trial on 20 ALS patients was conducted. In addition to findings of safety, a statistically significant indication of efficacy was found: patients taking l-serine at 5 and 15 g/day had a 22% reduction in the rate of functional decline, while those at 30 g/day of l-serine experienced an 85% reduction in functional decline by the slope of ALSFRS-R [21••].In January 2017, the FDA approved a Phase IIa clinical trial (IND 133607) of L-serine for Alzheimer's patients.

Given both the longevity and the apparent paucity of progressive neurodegenerative disease among the Ogimi villagers, an ethnobotanical study was designed to determine the most frequently consumed components of the Ogimi diet and to test those components for l-serine content.

## Methods

### Ethnobotanical Survey

The diet of Ogimi village, with a focus on the most frequently consumed food items, was studied through ethnobotanical surveys of the aged villagers in six different week-long expeditions to the village. Using participant observation techniques [22], villagers were initially surveyed to develop a comprehensive list of the 25 most common or highly salient food items. Villagers collecting marine algae or terrestrial plants were photographed, and the manufacture of tofu from soy beans by villagers was documented, as was the manufacturer of flour from the gametophytes of *Cycas revoluta* (Cycadaceae) which contains BMAA [23]. Interviews were conducted in Japanese but plant and animal species were referred to by their Ogimi names. Photographs of these food items were printed on 8 × 10 cardstock, one photograph per sheet, with the Ogimi name written in Hiragana above the photograph. Fifty different villagers were shown each photograph and questioned as to their personal frequency of consumption and the amount they consumed. Median frequency of consumption was then calculated for each food item. Samples of each food item were analyzed for amino acid content.

### Extraction of Food Items

Solid food items, obtained from Ogimi village, were placed into 50-ml plastic Falcon tubes and frozen at −80 °C. The sclerotesta from cycad seeds was removed and the gametophyte exposed before freezing in 50-ml Falcon tubes. After freeze-drying, subsamples were placed inside microcentrifuge tubes with the addition of 5–10 glass beads, then macerated using a bead beater (Omni bead beater 24, Omni International, Kennesaw, GA, USA) for 20–30 s. Once disrupted, a subsample was weighed into a 1.6-ml screw-capped glass vial. 6 M HCl was added at a concentration of 50 mg/ml. For liquid samples, 500 μl was placed into a 1.6-ml screw-capped glass vial and an equal volume of 12 M HCl was added. Samples were then hydrolyzed at 110 °C for 16 h. Once cooled, 200 μl of the hydrolysate were centrifuge filtered (0.22 μm; Millipore Ultrafree-MC, Billicore, MA, USA) and dried in a SpeedVac. Once dried, the residue was stored at −20 °C.

### Analysis of Collected Materials by Amino Acid Analyzer

Dried residues were re-suspended with 200 μl of 20 mM HCl and analyzed by amino acid analyzer (Hitachi 8900). Samples (10 μl) of the re-suspended extract or a 1/10 dilution with 20 mM HCl were injected into a Hitachi Amino Acid Analyzer L8900 equipped with a Hitachi Reaction column (PN 855-3533) at 135 °C, a high-speed physiological fluid analysis analytical column (Li-form resin #2622SC 6 mm ID × 40 L 060928C; PN855-4515), precolumn (PN855-3643), guard column #2619 4 mm ID × 5 L 2007.07.19 070639 (PN855-5268), and ammonia filter column (ion exchange 4.6 × 40 column #2650 L, PN 855-3523). Pre-made buffers (Hitachi) were used as follows: (B1) PF-1/AN0-8711, (B2) PF-2/AN0-8712, (B3) PF-3/AN0-8713, (B4) PF-4/AN0-8714, (B5) 5% methanol, (B6) PF-RG/AN0-8715, (R1) ninhydrin solution, (R2) ninhydrin-buffer of lithium acetate dihydrate, (R3) 5% ethanol. Separation was made with a flow rate for pump 1 of 0.53 and 0.45 ml/min for pump 2 and a 152 min gradient elution (0.0 min = 100% B1, separation column and guard column temp 35 °C, 50% R1, 50% R2; 1.5 min = column 32 °C; 15.6 min = 81% B1, 19% B2, column 57 °C; 36 min = 60 °C; 45 min = 32 °C; 57 min = 70 °C; 68 min = 15% B1, 75% B2, 10% B3; 69 min = 58 °C; 76.1 min = 60% B2, 40% B3; 89 min = 65 °C; 95 min = 20% B2, 80% B4; 98.1 min = 25% B2, 75% B4; 112.1 min = 100% B4; 125 min = 70 °C; 125.1 min = 100% R3; 129.1 min = 100% B6; 132.1 = 100% B1; 137.1 min = 50% R1, 50% R2, 35 °C).

Amino acid concentrations were determined from retention times of individual l-amino acid standards, corresponding to the 20 canonical amino acids (Sigma Chemical Co., St. Louis, MO). Using peak area and retention time, the amino acid concentrations were determined on a gravimetric basis for solid food samples and on a volumetric basis for liquid samples.

## Results

The top 25 most frequently consumed food items are listed in Table 1. Since dried bonito (*Katsuwonus pelamis*) is used as a seasoning in many indigenous dishes, it was arbitrarily placed at rank 25. It is notable that 13 species of marine algae occur in the top 25 most frequently consumed food items. Dietary preferences of the Ogimi diet showed only subtle variation between different ages of respondents. Comparing respondents between 50 and 79 years old with those 80 or older, older respondents ranked *Ipomoea batatas*, pork, and the marine algae *Gloiopeltis tenax*, *Codium yezoense*, and *Chondrus ocealltus* higher than did younger respondents.

**Table 1:** Amino acid profiles (mg/100 g) for marine algae and other frequently consumed dietary items in Ogimi village, Okinawa

	*Undaria*	*Saccharina*	*Sargassum*	*Gracilaria*	*Hypnea*	*Monostroma*	*Codium*	*Gloiopeltis*	*Glycine max*	*Diginea*	*Momordica*	*Moi dofu*	*Chondrus*	*Cycas*	*Ulva*	Bonito
Asp	1726	979	772	669	410	294	1199	837	3887	344	159	1159	170	1409	394	4931
Thr	857	459	379	375	293	143	575	517	1416	160	102	733	125	827	179	2290
Ser	771	392	340	254	197	139	513	353	1670	136	64	469	53	455	139	1910
Asn	ND	ND	ND	ND	ND	ND	ND	ND	ND	ND	ND	ND	ND	ND	ND	ND
Glu	1925	1597	982	648	471	356	1496	734	6168	284	199	1691	167	2004	651	6791
Gln	ND	ND	ND	ND	ND	ND	ND	ND	ND	ND	ND	ND	ND	ND	ND	ND
Cys	ND	149	53	30	77	20	100	23	ND	86	18	108	10	ND	60	ND
Pro	837	864	444	347	622	134	621	1083	4783	228	165	1432	195	1220	211	1945
Gly	744	341	316	272	181	114	488	467	1074	149	58	454	64	518	146	1716
Ala	1352	724	415	453	305	234	1019	476	1631	168	101	801	117	870	267	3334
Val	996	490	409	360	272	156	671	518	1478	142	98	756	92	813	197	2659
Met	386	171	129	ND	67	39	156	6	134	33	4	305	12	26	46	1452
Cystine	12	ND	ND	0	42	8	119	64	529	20	8	167	12	130	18	732
Ile	788	327	304	305	210	105	328	294	1350	119	75	664	67	663	142	2327
Leu	1468	587	550	511	312	199	822	549	2617	166	130	967	115	915	271	4124
Tyr	373	175	99	71	45	55	145	10	689	149	23	208	ND	132	8	1604
Phe	895	389	300	306	223	128	549	395	1756	162	79	581	81	502	182	2141
Trp	17	10	5	0	16	1	5	49	81	9	ND	7	ND	10	2	119
Lys	1114	428	322	258	263	128	613	430	2306	255	110	841	52	281	185	4699
His	460	155	112	187	73	52	224	248	1237	62	77	360	13	181	78	3378
Arg	1178	319	241	232	172	116	349	376	2519	87	136	461	60	1131	304	3476

*ND* not detected

**Fig. 1:**
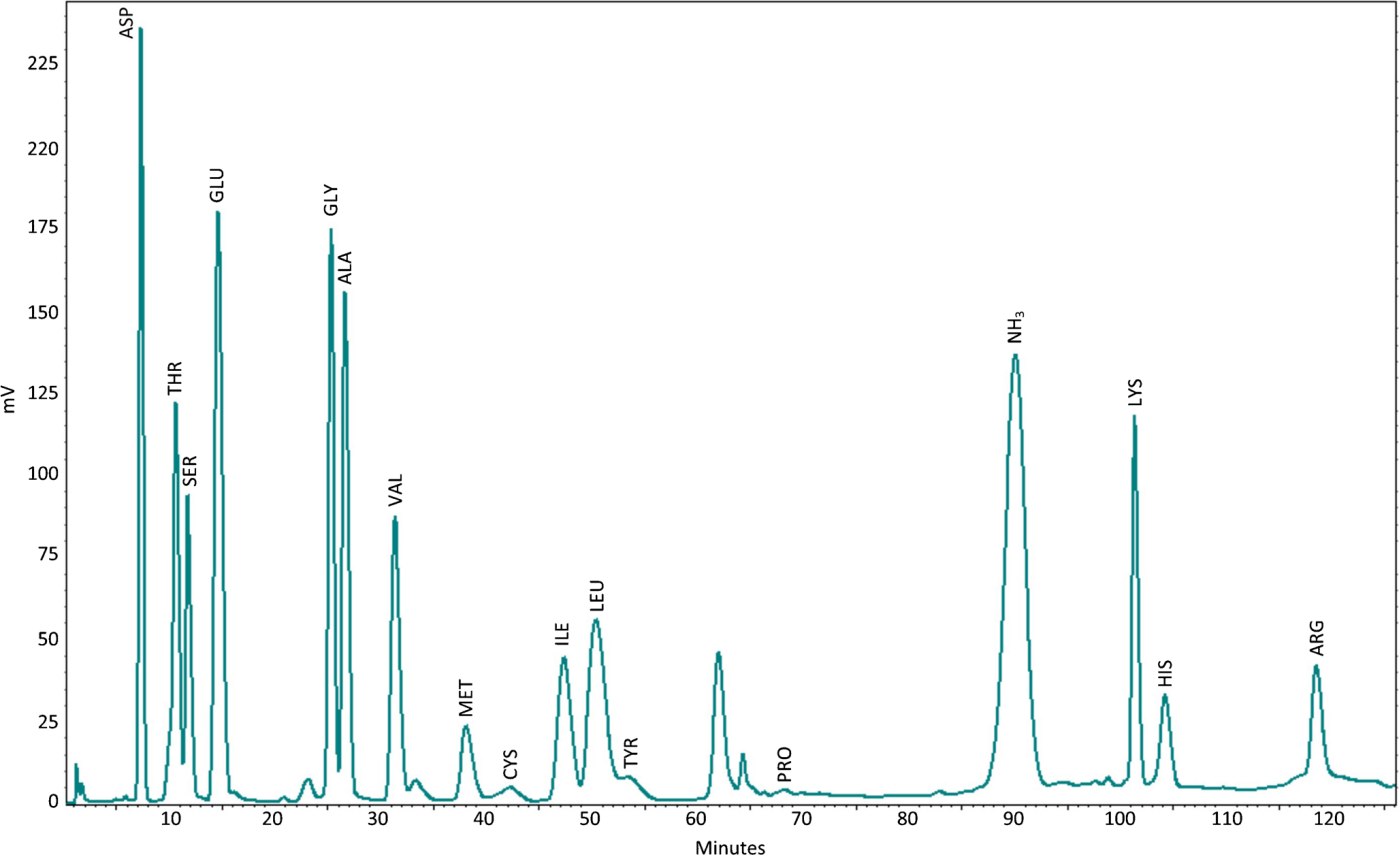
Amino acid profile of the marine algae *Hypnea charoides* (Cystocloniaceae) which had been cooked as moi dofu for consumption in Ogimi village, Okinawa, as determined by amino acid analyzer

Complete amino acid profiles of these frequently consumed items are shown in Table 2, with a representative chromatogram for the marine alga (*Hypnea charoides*, *moi* in the Ogimi language) cooked as *moi dofu* shown in Fig. 1. The Ogimi diet is remarkably rich in amino acids, including l-serine, with food items (excluding dried bonito) having a mean l-serine content of 542 mg/100 g. However, it does not appear that l-serine content alone impacts the relative frequency of consumption. The two most frequently consumed food items, *Citrus depressa* fruits (*kugani* in the Ogimi language) and rice, are relatively low in l-serine content. Neither the Spearman’s rank correlation coefficient for the l-serine content vs. rank of the top 25 preferred items (*r* = 0.15) nor for 13 taxa of marine algae in the top 25 items (*r* = 0.21) was significant at the 0.05 level. By weighing all food items consumed in a single day by an Ogimi villager and calculating from Table 2 the l-serine equivalents, it appears that total l-serine content of the Ogimi diet for women over the age of 70 is in excess of 8 g/day. This is about 6 g/day above the daily l-serine intake (2.53 g/day) from all sources consumed by women in the USA and twice the l-serine intake (7.15 g/day) consumed by the 99th percentile of US women age 71+ [24].

**Table 2:** Ranked frequency of consumption of the 25 top food items in the Ogimi diet compared to rank content mg/g of l-serine

Overall rank	Item	Ogimi name	Median	SER rank	Serine (mg/100 g)
1	*Citrus depressa* juice	*Kugani*	1	15	162.7*
2	*Oryza sativa*	*Gohan*	2	19	128.1
3	*Momordica charantia*	*Goya*	5	21	64.0
4	Tofu	*Tofu*	6	1	2351.5
5	*Undaria pinnatifida*	*Wakame*	8	5	771.4
6	*Saccharina japonica*	*Kombu*	9	10	391.5
7	*Glycine max*	*Edamame*	9	3	1670.3
8	*Ipomoea batatas*	*Beneimo*	10	8	480.0
9	*Ulva lactuca*	*Aasa*	10	17	138.8
10	*Artemisia princeps*	*Fuchiba*	12	4	805.3
11	*Sargasssum fusiformis*	*Hijiki*	12	12	339.5
12	Pork	*Samaniku*	12	2	1799.5
13	*Gynura japonica*	*Handema*	12	6	649.3
14	*Monostroma nitidum*	*Aasa*	13	16	138.9
15	*Hypnea charoides*	*Moi*	16	14	196.5
16	*Gracilaria vermiculophylla*	*Sunna*	17	13	253.9
17	*Cladosiphon okamuranus*	*Sunui*	17	20	74.1
18	*Gloiopeltis tenax*	*Funui*	18	11	353.3
19	*Chondrus ocealltus*	*Sinumata*	19	22	52.8
20	*Codium yezoense*	*Biru*	20	7	512.5
23	*Diginea simplex*	*Nachura*	21	18	135.9
24	*Cycas revoluta*	*Sotetsu*	22	9	455.4
25	*Katsuwonus pelamis*	*Kotsuo*	Unranked		

*mg/L

l-Serine-rich dietary items are consumed in religious festivals within Ogimi village, which occur several times a month. In the matriarchal society of Ogimi, women from the highlands bring *C. depressa* (*kugani*) fruits to these religious rituals, while coastal women bring marine algae. A meal is served consisting of tofu (23,510 μg/g l-serine), *Saccharina japonica* (*kombu*), and *H. charoides* (*moi*) which is boiled and mixed with bonito flakes and carrots or other vegetables, producing a gelatin-like substance called in Ogimi *moi dofu*. The amino acid profile of *moi dofu* (Fig. 1) shows that it is rich in l-serine and other amino acids.

## Discussion

**Fig. 2:**
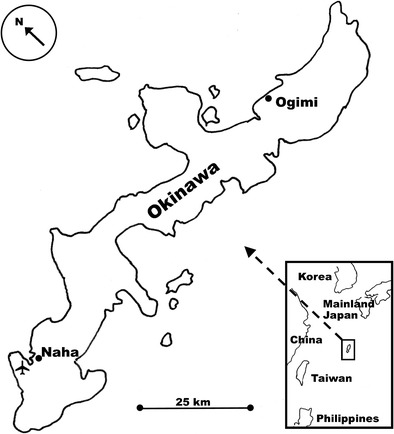
While politically part of Japan, Okinawa is remote from the Japanese mainland. Within Okinawa, Ogimi village is geographically, linguistically, and culturally isolated

Nutritional characteristics of food items consumed within Okinawa (Fig. 2) have received considerable attention over the last decade. Emphasis has been placed on the abundance of antioxidants in the food items as well as the low-calorie and high glycemic index of traditional Okinawan food [25]. Perhaps because of recent discoveries on the impact of caloric restriction on longevity of laboratory animals [26], emphasis has been placed on anti-obesity aspects of the Okinawan diet [2, 25, 27, 28], based on the assumption that caloric restriction will reduce risks of atherosclerosis and diabetes in human populations [29]. Indeed, post-war Okinawans with a diet of approximately 11% less than the 1949 mean daily caloric intake of 1785 kcal/day would have had a reduced body mass index (BMI)[2]. In our ethnobotanical surveys, Ogimi villagers often spoke of nutritional deprivation during and after World War II. The question is whether malnutrition of Okinawans during the war had a positive or deleterious impact [30]. However, as regards nutritional deprivation during war time, the Okinawans were not unique. Cox [31] in an analysis of height and weight measurements of nearly 600,000 German children in World War I found extensive indications of nutritional stress and highly reduced BMI as a result of chronic caloric deficits; however, there is no evidence of increased longevity among the German population, nor is there indication of increased longevity among other populations that have suffered caloric deficits as a result of war. We also note that in our ethnobotanical surveys, contemporary Ogimi villagers, while not obese, do not appear to have a significantly reduced BMI compared to other populations, and nothing approaching the 30–70% reduction in calories required in laboratory animals to increase longevity [32]. While not disputing the advantages of eating a plant-based diet, minimal tobacco use, and regular physical activity for a healthy lifestyle [33] which lower risks of cancer and type II diabetes [34], we suggest that a more careful consideration of the remarkable emphasis on soy products and marine algae in the Ogimi diet, particularly an analysis of the amino acid content of that diet, can give important clues as to the source of neurological health.

The importance of fermented soy products, in the form of tofu, and the frequency of consumption of *Glycine max* as edamame, which appear to be the major source of calories in the Ogimi diet (as opposed to rice in the rest of Japan), and particularly the importance and diversity of marine algae consumed by villagers, appear to distinguish the Ogimi diet. “The Okinawan people have always included a number of different varieties of seaweed in their everyday diet and in large amounts. *Konbu* [sic] is particularly used, which is not locally produced, nevertheless features in both traditional festival menus and in the everyday diet” ([35] pp. 163–164). The import of *kombu* (*Saccharina japonica*) from Hokkaido to Okinawa in general and Ogimi in particular stretches back nearly two centuries.

It is claimed that edible seaweeds in general, and *kombu* and *wakame* (*Undaria pinnatifida*) in particular, are good sources of protein [36, 37, 38] and offer a complete complement of essential amino acids [39, 40]. Although seaweeds are particularly rich in glutamic acid, alanine, and phenylalanine [41], we here note they are also rich sources of l-serine. *Hypnea charoides* and *Ulva lactuca* have been found by previous investigators to be good sources of l-serine [42, 43]. *Wakame* may also serve to reduce serum and lower triacylglycerol [44], while the fruits of *C. depressa*, which is the most frequently consumed food item in Ogimi village, have clear hypolipidemic effects, which together with the antioxidants present in the fruit reduce the risk of lifestyle disease [45].

## Conclusions

Food preferences and taboos embedded within indigenous religions are strong indicators of cultural importance [22, 46]. During frequent indigenous religious ceremonies in Ogimi, participants consume a meal which is extraordinarily high in l-serine. Although Ogimi villagers have not been aware either of the high amino acid content of their indigenous diet, nor of recent research suggesting that l-serine in particular has neuroprotective activity, both their religion and cultural practice ensure that they consistently receive one of the highest l-serine-containing diets in the world. The people of Ogimi are known to be long-lived. A wide range of potential factors could contribute to their longevity. Due to the unique nature of their diet, rich in seaweeds and tofu, the high serine content of their diet may offer neuroprotection and contribute to their neurological health in this community.
